# Supplementary material for: CD73-Mediated Immunosuppression Is Linked to a Specific Fibroblast Population That Paves the Way for New Therapy in Breast Cancer
Source: Cancers (Basel). 2021 Nov 23;13(23):5878. doi: 10.3390/cancers13235878 (PMC8657241; doi:10.3390/cancers13235878)
Supplement: Supplementary file 1 [file cancers-13-05878-s001.zip › cancers-1444750-supplementary.pdf]

Supplemental information for:

CD73-mediated immunosuppression is linked to a specific fibroblast population that paves the way for new therapy in Breast Cancer

Special Issue: Cancer-Associated Fibroblasts and Treatment Response
Guest editors, Dr. Paul Span and Dr. Marleen Ansems

Ilaria Magagna[1,2,3], Nicolas Gourdin[3], Yann Kieffer[1,2], Monika Licaj[1,2], Rana Mhaidly[1,2], Pascale Andre[3], Ariane Morel[3], Anne Vincent-Salomon[4], Carine Paturel[3] and Fatima Mechta-Grigoriou[1,2,*]

[1] Institut Curie, Stress and Cancer Laboratory, Equipe labellisée Ligue Nationale Contre le Cancer, PSL Research University, 26, rue d'Ulm, 75005 Paris, France
[2] Inserm, U830, Paris 75005, France
[3] Innate Pharma, 117 Avenue de Luminy BP 30191, 13276 Marseille, France
[4] Institut Curie, Hospital group, Department of Diagnostic and Theranostic Medicine, 75005 Paris, France

* **Correspondence:** Dr. Fatima Mechta-Grigoriou (ORCID Number: 0000-0002-3751-6989) Phone: +33 (0)1 56 24 66 53; Fax: +33 (0)1 56 24 66 50; E-mail address: fatima.mechta-grigoriou@curie.fr

SUPPLEMENTARY FIGURES LEGENDS

Supplementary Figure 1: CD73 total RNA and protein levels did not differ among BC subtypes

(**A**) CD73 protein levels in LumA (n=96), LumB (n=16) HER2 (n=54) and TN (n=65) BC, considering both epithelium and stromal compartments together (n=215). Boxplots are shown as median ±25%–75% quantiles. P-values from Mann-Whitney. (**B**) CD73 mRNA levels in BC patients from TCGA cohort (LumA (n=231), LumB (n= 125), HER2 (n=58) and TN (n=97) BC. P-values from t test with Benjamini-Hochberg correction for multiple testing. (**C**) CD73 mRNA levels in CAF subset fibroblasts isolated by sorting from BC and submitted to RNA sequencing (original data from (Costa, et al., 2018; Givel et al., 2018) (n=16 CAF-S1, n=4 CAF-S2, n=10 CAF-S4). Boxplots are shown as median ±25%–75% quantiles. P-value from pairwise Wilcoxon rank sum exact test. (**D**) Number of CD3+ T lymphocytes per mm^2 in LumA (n=22), HER2 (n=28) and TN (n=37) BC. Each dot represents one tumor (n=87). Data are represented as median. P-values from Mann-Whitney test (**E**) Number of FOXP3+ T lymphocytes per mm^2 in LumA (n=63), HER2 (n=30) and TN (n=56) BC. Each dot represents one tumor (n=149). Data are represented as median. P-values from Mann-Whitney test.

Supplementary Figure 2: CAF-S1 do not modify CD73+ and CD39+ content in CD4+ CD25+ FOXP3+ Tregs

(**A**) Phase contrast images of 3 representative primary CAF-S1 cell lines isolated from 3 different patients prior to the 10th passage. (**B**) Representative contrast microscopy images of CD4+ CD25+ T cells alone (left) or co-cultured with CAF-S1 (right). (**C**) Flow cytometry plots showing the proportion of CD39+/- among CD4+ CD25+ FOXP3+ T lymphocytes either alone (left) or in presence of CAF-S1 (right). (**D**) Percentages (%) of CD39+ FOXP3+ (left), CD39+ $FOXP3^{low-med}$ (middle), CD39+ $FOXP3^{high}$ (right) T cells among CD4+ CD25+ T cells alone or upon co-culture with CAF-S1. Each dot represents one donor (n=16). 5 CAF-S1 independent primary cell lines tested. P values from paired t test for the proportion of CD39+ FOXP3+. P-values from paired Wilcoxon test for the proportion of CD39+ $FOXP3^{low-med}$ and CD39+ $FOXP3^{high}$. (**E**) Flow cytometry plots representing the proportion of CD73+ FOXP3+ among CD25+ FOXP3+ T cells from healthy donors in presence of CAF-S1. (**F**) Same as (**D**) for CD73. Each dot represents one donor (n=11). 4 CAF-S1 independent primary cell lines tested. P-values from Wilcoxon test for the proportion of CD73+ FOXP3+ and CD73+ $FOXP3^{high}$. P-values from paired t test for the proportion of CD73+ $FOXP3^{low-med}$.

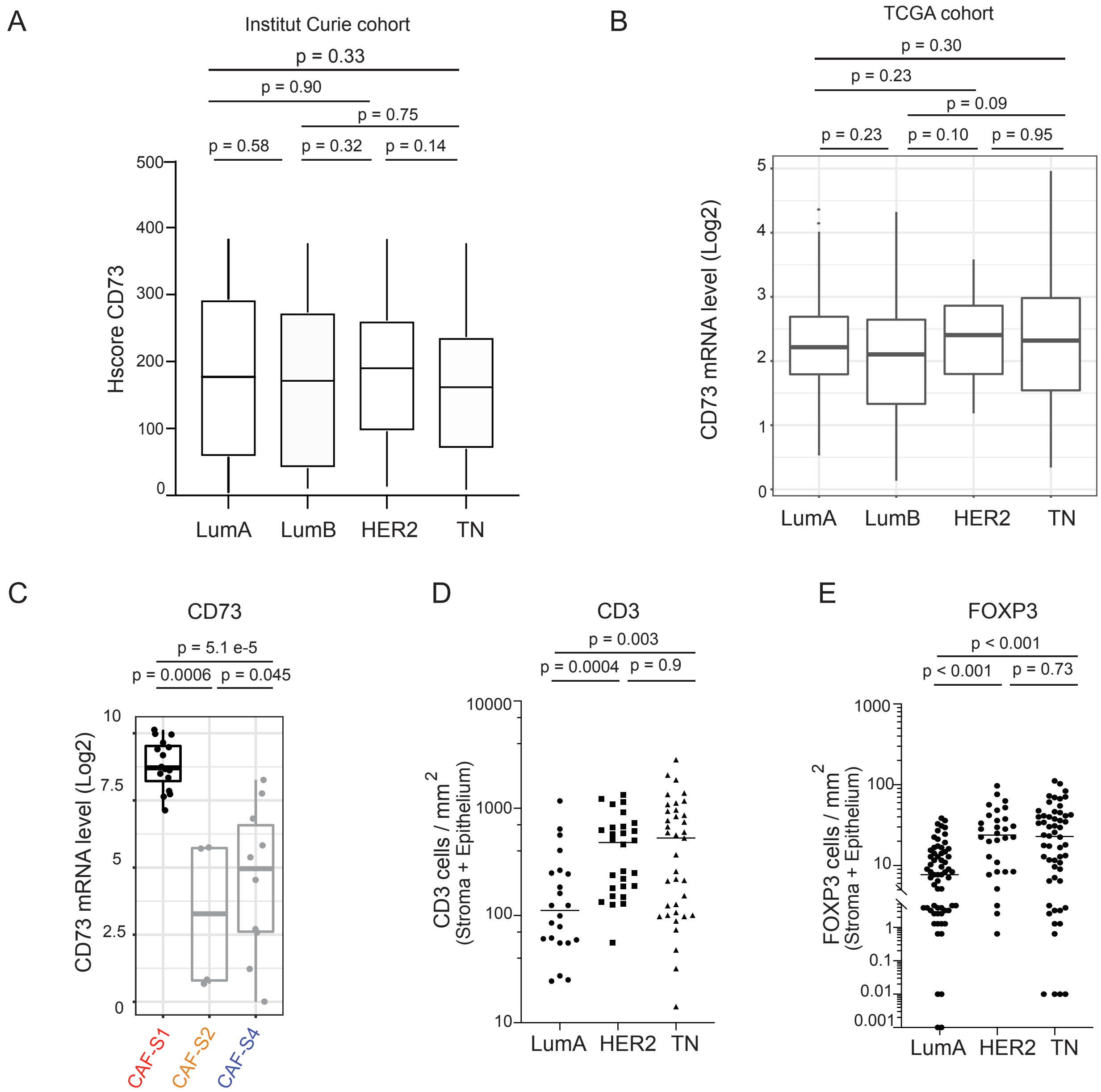
A
Institut Curie cohort
p = 0.33
p = 0.90
p = 0.75
p = 0.58
p = 0.32
p = 0.14
Hscore CD73
LumA
LumB
HER2
TN
B
TCGA cohort
p = 0.30
p = 0.23
p = 0.09
p = 0.23
p = 0.10
p = 0.95
CD73 mRNA level (Log2)
LumA
LumB
HER2
TN
C
CD73
p = 5.1 e-5
p = 0.0006
p = 0.045
CD73 mRNA level (Log2)
CAF-S1
CAF-S2
CAF-S4
D
CD3
p = 0.003
p = 0.0004
p = 0.9
CD3 cells / mm2 (Stroma + Epithelium)
LumA
HER2
TN
E
FOXP3
p < 0.001
p < 0.001
p = 0.73
FOXP3 cells / mm2 (Stroma + Epithelium)
LumA
HER2
TN

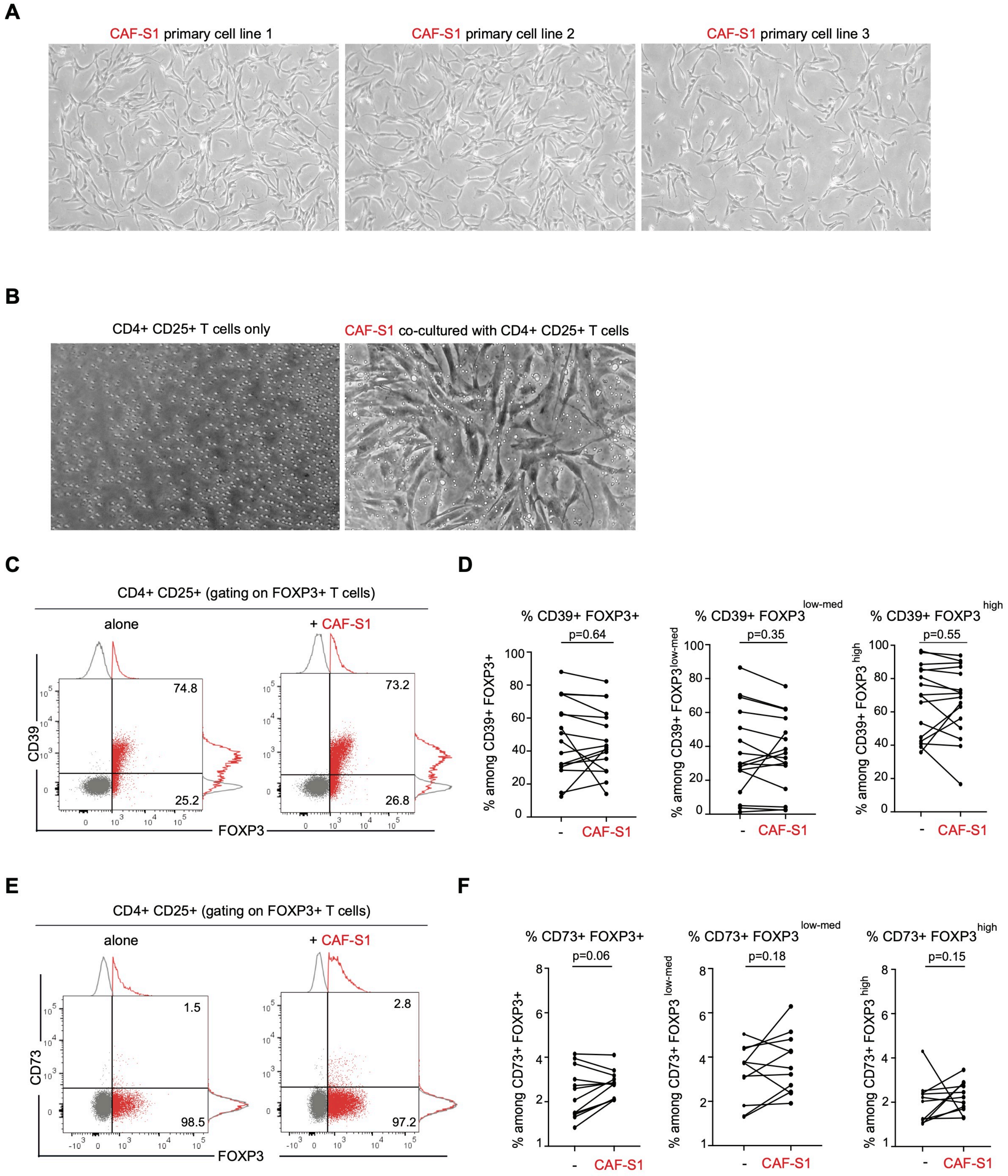
A
CAF-S1 primary cell line 1
CAF-S1 primary cell line 2
CAF-S1 primary cell line 3
B
CD4+ CD25+ T cells only
CAF-S1 co-cultured with CD4+ CD25+ T cells
C
CD4+ CD25+ (gating on FOXP3+ T cells)
alone
+ CAF-S1
74.8
25.2
73.2
26.8
CD39
FOXP3
D
% CD39+ FOXP3+
% CD39+ FOXP3 low-med
% CD39+ FOXP3 high
p=0.64
p=0.35
p=0.55
% among CD39+ FOXP3+
% among CD39+ FOXP3 low-med
% among CD39+ FOXP3 high
- CAF-S1
E
CD4+ CD25+ (gating on FOXP3+ T cells)
alone
+ CAF-S1
1.5
98.5
2.8
97.2
CD73
FOXP3
F
% CD73+ FOXP3+
% CD73+ FOXP3 low-med
% CD73+ FOXP3 high
p=0.06
p=0.18
p=0.15
% among CD73+ FOXP3+
% among CD73+ FOXP3 low-med
% among CD73+ FOXP3 high
- CAF-S1
